# Mapping the human thalamus: a historical review of thalamic atlases from histology to modern neuroanatomy

**DOI:** 10.3389/fnana.2026.1927141

**Published:** 2026-08-10

**Authors:** Michelle Antonios, Gabor Szekely, Theofanis Karayannis, Andras Jakab

**Affiliations:** 1 Laboratory of Neural Circuit Assembly, Brain Research Institute, University of Zurich, Zurich, Switzerland; 2 Center for MR Research, University Children’s Hospital Zurich, Zurich, Switzerland; 3 URPP Adaptive Brain Circuits in Development and Learning (AdaBD), University of Zurich, Zurich, Switzerland; 4 Computer Vision Laboratory, ETH Zurich, Zurich, Switzerland

**Keywords:** brain atlas, cytoarchitecture, diffusion tractography, MRI, thalamic nuclei, thalamus

## Abstract

This review outlines the development of thalamic atlases from early post-mortem 2D histological maps to modern 3D multimodal atlases used in neuroscience and clinical practice. Given the central role of the thalamus in sensory processing, movement, and cognition, accurate anatomical mapping has been essential for both research and neurosurgical targeting. By synthesizing historical and modern approaches, we highlight the methodological advances, such as the development of thalamus-specific MRI sequences, that have improved the precision of thalamic nuclei delineation. We focus on open-source atlases that support reproducible research and clinical applications. Future developments will likely focus on integrating multi-scale data; combining cytoarchitectonic, with quantitative MRI and computational models to improve thalamic parcellation and accurate localization *in vivo*.

## 1 Introduction

The thalamus is a bilateral structure located anterior to the brainstem, in the dorsal part of the diencephalon, making up 80% of its volume. Structurally, the thalamus is subdivided into multiple nuclei, each characterized by distinct cytoarchitecture, connectivity, and function. Traditionally, the thalamus has been regarded as a relay center for sensory and motor inputs (Guillery and Sherman, 2002). Neuroimaging studies have demonstrated that it plays an important role (Sherman, 2007; Rikhye et al., 2018) in higher-order functions, including attention, working memory, cognition, consciousness and perception (Hwang et al., 2021, 2022; Seo et al., 2022). Its multifaceted role is supported by its wide-spread connectivity with various structures in the CNS, as demonstrated across different imaging modalities mainly using resting state-fMRI (Hwang et al., 2017; Steiner et al., 2020), and probabilistic tractography in humans and high-resolution anatomical studies in other species (Behrens et al., 2003; Lambert et al., 2017). Most thalamic nuclei receive afferent inputs from subcortical structures including the spinal cord, basal ganglia and retina, while reciprocal connections are established with the cerebral cortex, amygdala and thalamic reticular nucleus. In addition, several nuclei, such as parafasicular and centromedian nuclei, give rise to projections to the striatum, as shown in rodent research (Berendse and Groenewegen, 1990; Mandelbaum et al., 2019). This distinctive connectivity pattern places the thalamus in a unique position to modulate large scale brain dynamics and contribute to many cognitive and behavioral functions (Shine et al., 2023).

Historically, one of the greatest challenges in neurosurgery was safe access to deep brain structures like the thalamus without damaging surrounding tissue and potentially causing irreversible damage to the patient. The development of stereotactic neurosurgery in humans in the mid-20th century, pioneered by Spiegel et al. (1947) based on earlier work by Horsley and Clarke (1908) represented a shift. By introducing a 3D coordinate system, stereotaxy enabled minimally invasive targeting of thalamic nuclei for therapeutic interventions such as lesioning and later deep brain stimulation (DBS). However, early stereotactic procedures relied on indirect targeting, as deep structures were invisible on conventional neuroimaging. With the development of MRI and progressive improvements in resolution and contrast, neurosurgeons and neuroscientists gained access to *in vivo* visualization of thalamic anatomy. Yet, even modern MRI has limitations in distinguishing many thalamic nuclei, highlighting the enduring importance of detailed brain atlases (Coffey, 2009).

“Cerebral cartography” or map-making has been an important endeavor of neuroscience, and in this process, numerous brain atlases have been created. A brain atlas is a comprehensive representation of the structure and, increasingly, the function of the brain. Atlases provide a common spatial reference that allow alignment of individual brain images, comparison across subjects and integration of multimodal data. By labeling and delineating distinct brain regions and structures, atlases support investigations of brain functions, guide neurosurgical planning and facilitate lesion-symptom mapping. Digital brain atlases can be broadly subdivided into two types: deterministic (single subject) or probabilistic. Deterministic atlases rely on high quality segmentation of an individual brain, whereas probabilistic atlases combine data from multiple subjects, co-registering all the segmented regions to a standard space and computing the frequency of each voxel to belong to a specific region.

Early atlases were based on histological sections, carefully reconstructed in two dimensions. Although these resources remain the gold standard for cytoarchitectonic delineation, they are inherently limited by deformation, shrinkage, and the absence of individual variability. Advances in computational methods have allowed these histological datasets to be digitized, reconstructed in three dimensions, and registered to MRI templates (Chakravarty et al., 2006; Iglesias et al., 2018; Alkemade et al., 2022). Meanwhile, MRI-based atlases, using sequences optimized for intra-thalamic contrast, as well as diffusion-based connectivity profiles, have enabled *in vivo* parcellation. The two strategies offer complementary strengths: histology offers microscopic precision, and MRI ensures clinical applicability.

Human brain atlases were historically distributed in print, but the digital advances have since enabled interactive, continuously updated, and openly accessible platforms (Nowinski, 2021). Modern atlases are often accompanied by analysis pipelines that allow automated segmentation in individual subjects (Ding et al., 2016; Amunts et al., 2020; Alemán-Gómez et al., 2022; Alkemade et al., 2022). 3D atlases of the thalamus have emerged as an essential tool for our understanding of thalamic topography (Morel et al., 1997), support localization of functional MRI responses, guide functional neurosurgical interventions (Morel et al., 1997; Niemann et al., 2000; Martin et al., 2009; Krauth et al., 2010) and enable lesion-symptom mapping studies.

**FIGURE 1:**
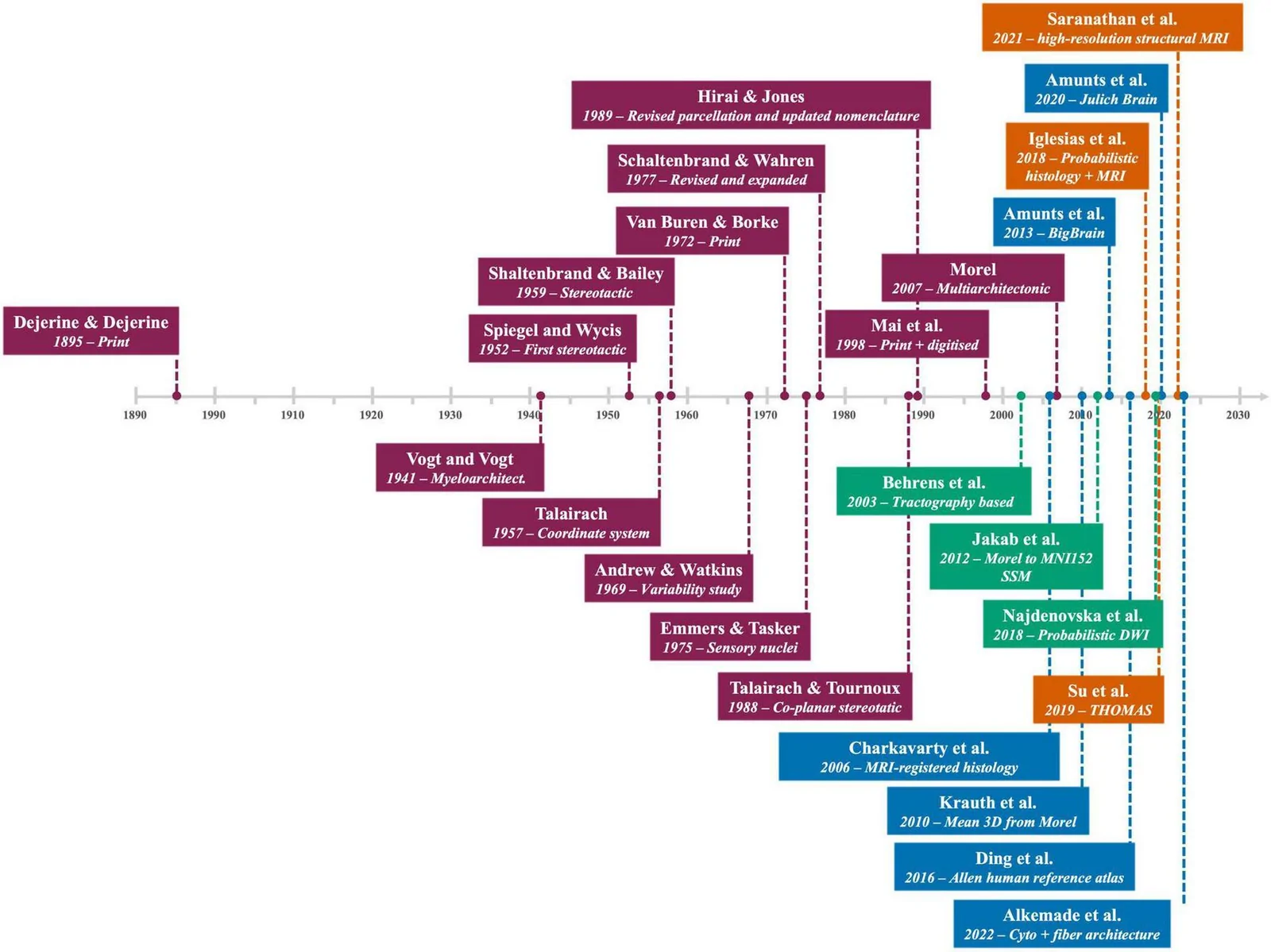
Timeline of human thalamic atlas development, from print and stereotactic atlases (purple) to digitized histology-based reconstructions (blue), connectivity and diffusion MRI-based parcellations (green), and structural MRI atlases (orange).

The aim of this review is to provide an overview of thalamic atlases, tracing their historical development from early stereotactic and histology-based approaches to modern MRI-based and multimodal methods. We highlight the methodologies of atlas construction, the imaging methods that have enabled intra-thalamic delineation, and the software platforms that incorporate these resources. By synthesizing this information, our goal is to offer a clear reference for neuroscientists, neuroimagers, and clinicians seeking to select and utilize thalamic atlases for research, functional localization, and clinical applications. We included studies describing the construction of human thalamic atlases, parcellation frameworks, or mapping datasets. Textbooks and historical atlases available only in print format were identified through supplementary manual searches and reference screening and were included where they represented significant methodological milestones. Figure 1 summarizes the historical trajectory of thalamic atlas development, illustrating the major methodological shifts from classical anatomical descriptions through stereotactic frameworks to modern probabilistic and multimodal approaches.

## 2 A review of histology-based atlases

Histological atlases represent the foundation of neuroanatomical research. Developed from cellular-resolution microscopy data, such atlases present findings as mesoscopic representations of brain structures emphasizing the cytoarchitectonic organization. They can be broadly categorized into traditional book-format stereotactic atlases and modern digital atlases, reflecting the field’s evolution from physical print volume to computationally accessible datasets. Table 1 summarizes the major histological atlases developed over the past two decades, from early digitized approaches to modern high-resolution datasets.

**TABLE 1:** Characteristics of histology-based digital thalamic atlases.

Atlas (year)	Chakravarty et al., 2006	Morel, 1997/2007; Krauth et al., 2010	BigBrain, 2013	Ding et al., 2016	Iglesias et al., 2018	Julich-Brain Atlas, 2020	Alkemade et al., 2022
Dimension	3D	2D histology + 3D reconstruction	3D	2D histology + 3D reconstruction	3D (probabilistic)	3D	3D
Resolution	∼0.5–1 mm (MRI-based)	∼1 mm (reconstructed)	20 μm isotropic	1 μm in-plane	∼1 mm (*in vivo* MRI)	20 μm (histology); ∼1 mm (MNI output)	20 μm (sections); 200 μm (3D volume)
Sectioning	MRI-based	Coronal, sagittal, horizontal	Coronal (reconstructed to 3D)	Coronal	Coronal (*ex vivo* histology)	Coronal	Coronal
Parcellation	Subcortical structures (thalamus + basal ganglia)	Thalamus + basal ganglia (42 nuclei)	Whole brain	Whole brain	Thalamus only (26 nuclei)	Whole brain (probabilistic cytoarchitectonic maps)	Whole brain nuclei + fiber tracts
Staining /contrast	Nissl, Luxol Fast Blue (myelin)	Luxol Fast Blue (myelin) Nissl, AChE, CR, CB, PV, SMI-32	Modified Merker (cell bodies)	Nissl, SMI-32, PV, AChE, MRI, DWI	Nissl (histology) + *ex vivo* MRI	Nissl, IHC, receptor mapping	Nissl, Bielschowsky silver (fibers), PV, CR, CB + 7T MRI
Samples (# brains)	1	6	1	1	6 (*ex vivo* histology) + 39 (*in vivo* MRI)	23	2
Coordinate system	Colin27	AC–PC aligned → MNI	Histology → MNI	Atlas-specific space	Bayesian inference → *in vivo* MRI (FreeSurfer)	MNI probabilistic space	Blockface → MRI

MRI, magnetic resonance imaging; IHC, immunohistrochemistry; CR, calretinin; CB, calbindin; PV, parvalbumin; AChE, acetylcholinesterase; SMI-32, non-phosphorilated neurofilament protein; AC, anterior commissure; PC, posterior commissure; DWI, diffusion weighted imaging.

### 2.1 Classical 2D atlases

The first systematic, histological, and clinically informed depiction of the human thalamus was published in 1895 by Jules and Augusta Dejerine in their book “Anatomie des center nerveux” (Déjerine and Déjerine-Klumpke, 1895). Their work used a multi-modal approach using serial sectioning of the brain in coronal, axial and sagittal axes. They used histological labeling such as Nissl staining (for neuronal cell bodies and gray matter organization), Weigert staining (for myelinated fibers and white matter tracts) and Golgi method (to some extent, for individual neuron morphology). While their work did not specifically identify and label individual nuclei according to modern thalamic nomenclature, they did segregate the thalamus into large zones (e.g., internal, median and external nucleus, pulvinar) based on cell density, fiber orientation, and laminar organization. Pascal et al. (2018) mapped the thalamic structures from the Dejerine atlas text to the BigBrain dataset, in an effort to enable a 3D visualization of thalamic nuclei, and to align the terminology used in the 20th century with the modern thalamic nomenclature.

Another pioneering work for understanding the anatomy and characteristics of the thalamus was published by Vogt and Vogt (1941). The authors hypothesized that the thalamus may be subdivided by “sharp” linear boundaries between nuclei and critically examined whether the fiber lamellae truly represent such divisions, as described by earlier authors. Using multiple specimens, they applied s-, cyto-architectonic and fiber-connection tracing methods, to study the distribution and architecture of neuronal populations and myelinated fibers of the thalamus, as well as their connections and boundaries. The publication shows hand-drawn, anatomically accurate plates based on their microscopic observations, in addition to detailed topographical maps integrating findings from serial sections. While their article discusses the whole thalamus, their actual work focuses on the centromedian nucleus. Their work became foundational for understanding functional-anatomical organization of the thalamus and provided a framework still recognizable in current thalamus nomenclature, despite later revisions and enhancements.

### 2.2 Stereotactic 2D atlases

The work of Spiegel and Wycis (1952) has provided one of the fundamental atlases of the human thalamus created for the purpose of the precise delineation of nuclei for surgical procedures. The book features a series of coronal photographs of a post-mortem brain, sliced in relation to the posterior commissure (PC) and the midline. To aid in identification of structures, they authors included myelin stainings, as well as their atlas includes a variability table to assess the differences measured across 30 brain specimens (Lehman and Augustine, 2013). By referencing distances in millimeters from the PC, their method allowed surgeons to better localize deep brain targets, bypassing the “fixed” atlas coordinates or external skull landmarks. The atlas labels several thalamic nuclei, including the anterior, ventrolateral, and dorsolateral nuclei. This work marked a shift from descriptive to interventional neuroanatomy, laying the foundation for modern functional neurosurgery in movement and psychiatric disorders.

Talairach et al. (1957) introduced a new coordinate system, known as Talairach’s coordinate system that uses the anterior commissure (AC) and posterior commissure (PC) as intracerebral stereotactic markers. To localize the AC-PC, the authors used positive contrast and air ventriculography, methods used prior to modern imaging methods (i.e., MRI or CT). In a single post-mortem brain, air was injected into the ventricular system to provide radiographic contrast on X-ray images, making the internal structures visible and enabling the identification of the AC-PC. Calculating the AC-PC distance provides a reference framework for comparing brain structure locations across different individuals. The AC-PC system has been the gold standard for brain mapping, essential for surgery and for correlating clinical and anatomical data in both research and clinical contexts for several decades. Talairach’s atlas included thalamic nuclei maps and other structures in a 3D profile along with a technique to identify the ventral layer of thalamic nuclei. This coordinate framework was later extended by Talairach and Tournoux (1988). The presented a new “referencing space” to address interindividual anatomical variability. Unlike the 1957 system, this space is based on “orthogonal parallelograms” or voxels representing a fixed fraction rather than absolute distances within the brain. The reference planes are based on the midline, the intercommissural plane and two verticofrontal planes intersecting the AC and PC. Each hemisphere is subdivided into 9 major sections in length along the IC line, 4 sections in width along the transverse plane orthogonal to the midline and IC plane, and 12 in height parallel to the planes defined by the commissures. While this grid system does not describe in detail the thalamic nuclei, it became a valuable framework for planning and analyzing surgical approaches for deep brain structures using stereotactic techniques.

The Schaltenbrand and Bailey atlas, published in 1959, was based on 111 brains sectioned in the coronal, sagittal and horizontal planes, employing various staining methods (unstained, stained for myelin or cresyl violet) (Schaltenbrand and Bailey, 1959). Among these, seven brains underwent comprehensive analysis. Their coordinate system was largely based on Talairach’s space with some differences. This atlas depicted the thalamic somatosensory relay nucleus which proved essential in guiding thalamotomies for tremors of the upper extremities.

To address the differences across- and within-individuals in thalamic and adjacent subcortical structures, particularly due to the limited precision of stereotactic coordinates and inter-hemispheric asymmetries, Andrew and Watkins (1969) conducted a morphometric and stereotactic variability study. Their work consisted of serial coronal sectioning of 38 cerebral hemispheres at 1 mm intervals, oriented perpendicular to the foramen of Monro - posterior commissure (F.M.–P.C.) plane. Boundaries of thalamic nuclei were measured relative to both the midline and F.M.–P.C. plane with anterior-posterior limits normalized using the foramen of Monro as a common reference point. The atlas includes major thalamic nuclei, adjacent diencephalic regions, basal ganglia, and major white matter tracts. It provides photographs and line drawings accompanied by statistical summaries, probability tables and distances to key landmarks. The stereotactic coordinate system was defined by the F.M.–P.C. plane and the total thalamic length (from foramen of Monro to the pulvinar tip), allowing for consistent intra- and intersubject comparison.

In an effort to update and unify thalamic nomenclature to better reflect the variability observed in the human brain, Van Buren and Borke (1972) an atlas detailing the cytoarchitecture of the individual thalamic nuclei and their connections. They were interested in identifying the thalamic variations for localization of recording and stimulation points during stereotaxic surgery, and more broadly, in producing a human-specific account of thalamic organization, given the inadequacy of animal-derived atlases for clinical use. In their work, the authors reviewed the literature for the nuclei they investigated and compared it to their own. Using the classical staining methods (cresyl violet, Golgi and myelin staining), they used the cells’ shape, staining affinity and size to characterize the nuclei. This systematic cytoarchitectonic characterization allowed them to address a central problem in thalamic nomenclature: the difficulty in having a unified nomenclature when the methods for data collection arise from different methodologies and sometimes originate from different species (Powell, 1973). By grounding their parcellation in the histological morphology of neuronal component, they let the patterns guides reconstruction rather than adhering strictly to a single nomenclature tradition. They generally followed the terminology of the German school of Vogts and Hassler (Vogt and Vogt, 1941; Schaltenbrand and Bailey, 1959) but introduced divergencies and provided a table of equivalents to facilitate comparison with major previous descriptions (Powell, 1973). To infer connectional pathways, the authors examined postmortem human brains with known lesion histories, identified which thalamic nuclei underwent retrograde degeneration following damage to specific cortical regions. The atlas is constructed based on serial sections in multiple planes (coronal, sagittal, and horizontal), allowing detailed anatomical and connectional mapping, with each plane annotated for nuclear boundary identification and connectional pathways. The work also relates the observed anatomical clusters to functional domains, bridging the gap between structure and connectivity and provides anatomical references for clinicians.

Emmers and Tasker (1975) introduced an atlas of the “somesthetic” thalamus, meaning the group of thalamic nuclei responsible for relaying sensory input, such as the ventral posterolateral and ventral posteromedial nuclei. Their atlas included whole brain photographs and microscopic sagittal and coronal sections from another brain (Emmers and Tasker, 1975). The site of electrical stimulations is illustrated with the type and distribution of somesthetic responses evoked during surgery, providing functional maps for intraoperative targeting during stereotactic procedures of the sensory thalamus.

The abovementioned atlases are all out of print at the time our review was written. The atlases described below are the traditional stereotactic atlases that are still in print.

Schaltenbrand and Wahren (1977) developed one of the most used thalamic atlases clinically, for functional neurosurgery. The atlas was produced through detailed histological sectioning from two brains oriented according to the AC-PC reference system and cut in the frontal, sagittal, and horizontal planes. The atlas presents macro representations from unstained sections and micro representations from myelin-stained sections. Some sections were photographed at a macro level unstained, and some were myelin stained to enhance delineation of subcortical structures such as the basal ganglia and thalamus and photographed at high magnification with overlays for measurement and labeling. The resolution of the atlas was constrained by its sectioning density, with frontal slices spaced 1–4 mm apart, which is sparse compared to modern standards (Conti et al., 2023). It was later digitized reconstructions interpolated these data into ∼0.5 mm isotropic voxels. This marks the first computerized atlas digitization (Sadikot et al., 2011). Furthermore, the Schaltenbrand-Wahren atlas relies on fixed distances from the intercommissural line, instead of the AC-PC points. As it relies on one point only in the A-P axis, scaling it to individual brains is not as precise as the AC-PC coordinate frame. Despite these limitations, the atlas’ combination of unstained macro-series and myelin-stained micro-series improved the anatomical depiction of the thalamus, and this atlas remains a standard reference to date. The Schaltenbrand and Wahren stereotaxic atlas adopts the thalamic nuclear terminology and parcellation scheme developed by Hassler (1982), identifying 60 distinct thalami nuclei. A key contribution of the atlas, at the time, is a more detailed representation of the thalamus, including overall anatomy and subdivisions of the thalamic nuclei, which are presented in all three anatomical planes. This atlas has been extensively used in both clinical and research contexts for stereotactic targeting. Although neuroimaging studies confirm that these delineations can be used for surgical localization, spatial inconsistencies between planes and inaccuracy in 3D localisation might arise from histological distortions and reliance on a limited number of samples (Sramka et al., 1998; Niemann and van Nieuwenhofen, 1999; Nowinski, 1999; Nowinski et al., 2008).

Hirai and Jones (1989) addressed a long-standing problem in thalamic nomenclature. Human thalamic anatomy was still described using Hassler’s (1982) terminology, which differed substantially from the nomenclature used in non-human primates, making it difficult to relate human finding to extensive experimental literature on thalamic connectivity and function. This discrepancy became increasingly important with the resurgence of stereotactic thalamotomy and the growing use of intraoperative microelectrode recordings, whose interpretation at the time relied heavily on data form non-human primates. To address this, Hirai and Jones examined six post-mortem human thalami, using serial histological sections stained for Nissl substance, myelin, cytochrome oxidase and acetylcholinesterase (AChE). AChE staining was particularly useful for delineating the internal medullary lamina and its associated nuclei. This revealed a histochemical organization closely matching that of the macaque thalamus. Based on these findings, the authors proposed a revised parcellation that adopted the nomenclature of the monkey thalamus and aligned human nuclear boundaries with a framework supported by decades of experimental work. This involved renaming the nuclei and redefining several subdivisions, such as the ventral nuclear complex. More broadly, this study highlights a defining feature of human thalamic research: much of our understanding of thalamic organization and function still relies on evidence from invasive studies in experimental animals, particularly non-human primate. Because such approaches are not feasible in living humans, cross-species comparisons remain essential for interpreting human anatomy and physiology.

The “Atlas of the Human Brain,” first released in 1997, is available in both print and digital formats (Mai et al., 1997), and it is the first atlas to combine MRI with histology. The latest edition (Mai et al., 2015), published in 2015, consists of two complementary atlases. The first is a macroscopic atlas presenting MRI scans of three brains in three coordinate systems. These brains were sectioned in 1-cm thick slices, which displayed alongside the MRI images, anatomical drawings with labels, and X-ray radiographs illustrating vascular territories. The second is a myeloarchitectonic atlas based on a right cerebral hemisphere sectioned coronally and stained by Oscar and Cécile Vogt. It contains 99 myelin-stained sections paired with corresponding anatomical diagrams, as well as 50 cell-body-stained sections interposed at half the frequency of the myelin series. The sections are adjusted to MNI space (Mazziotta et al., 1995, 2001). The final part of the atlas includes high-magnification maps of subcortical structures, including the thalamus, with detailed delineations by F. Forutan.

Several atlases are specific for the human thalamus. The stereotactic atlas of the human thalamus and basal ganglia, published by Morel (2007), represents a high-resolution cytoarchitectonic and chemoarchitectonic reference designed to refine stereotactic localization of the human thalamus, basal ganglia and bordering structures. Importantly, building upon the nomenclature proposed by Hirai and Jones (1989), this atlas also introduced a revised nomenclature and a refined parcellation of thalamic nuclei, based on combined cytoarchitectonic and chemoarchitectonic criteria. The Morel atlas largely retains the core nomenclature of Hirai and Jones, while introducing finer subdivisions for certain nuclei, such as the mediodorsal (MD), subdivided into MDmc, MDpc, and MDpl, and the ventral lateral nucleus (VL), subdivided into VLpv, VLpd, and VLpl. In addition, Morel’s chemoarchitectonic maps define functional territories that cut across traditional nuclear boundaries. The Morel atlas includes figures from serial histological sections of post-mortem human brains in the three stereotactic planes. The sections are stained with classical Nissl and myelin, and a range of immunohistochemical markers, including calretinin, acetylcholinesterase, calbindin, and parvalbumin. The atlas complements its histological plates with corresponding MRI of the same brains, aligned to the AC-PC plane. These MRI images provide macroscopic landmarks that help registration of the atlas to *in vivo* imaging. By integrating multiple specimens and applying alignment methods, the atlas reduced histological distortions and improved three-dimensional consistency, making it more suitable for stereotactic applications. Although still constrained by postmortem variability and histological artifacts, the Morel Atlas remains the most comprehensive and widely used reference for the human thalamus and basal ganglia in both clinical and research contexts, and is the basis of several 3D, digital atlases of thalamus, as further detailed in our review.

A recent 2D atlas, published by Ding et al. (2016), is exclusively available in electronic format. The atlas delivers an unprecedented digital reference of the entire adult human brain at cellular resolution by combining multimodal imaging, high-resolution histology, and detailed structural annotation. It encompasses structural MRI data, diffusion-weighted imaging (DWI), and 1,356 large-format histological plates, both Nissl and immunohistochemically stained, captured at a microscopic resolution of 1 μm per pixel. Anatomical delineations comprise 862 brain structures, including 117 white matter tracts and many novel cytoarchitectonic and chemoarchitectonic parcellations, all mapped and transposed onto the corresponding MRI volumes. Sampling was selective: 106 representative whole-hemisphere sections were annotated using intervals ranging from 0.4 mm (for smaller subcortical areas) up to 3.4 mm (in anterior/posterior cortical zones). Due to the high resolution and dense Nissl-sampling, the authors describe a novel subdivision of the mediodorsal nucleus (MD) of the thalamus. This subdivision, named anteromedial subdivision of the MD, exhibits enrichment in acetylcholinesterase (ACHE) and neurotensin (NTS) in comparison to the main MD portion. The atlas is accessible online, integrated with the Allen Institute’s gene expression datasets, and features an interactive, multiscale tool, from macroscopic MRI landmarks to histology.

### 2.3 3 D atlases

Although many 3D atlases have been developed based on printed 2D atlases (Yoshida, 1987; Nowinski et al., 1997; St-Jean et al., 1998; Sudhyadhom et al., 2012), this review focuses on atlases developed from original data acquisition. The pioneering effort in this field came from the Montreal Neurological Institute (MNI), Canada (Chakravarty et al., 2006). Their rationale to develop this atlas was due to the limited inherent resolution in the slice direction axis, with a limited number of structures and the few mis-registrations between the digital atlas and the Colin27 MRI average reference that are found in the digital atlas developed at the MNI by St-Jean et al. (1998). In their atlas, Chakravarty et al. (2006) used serial Nissl and myelin-stained coronal sections of the basal ganglia and thalamus from a single subject, which were digitized and manually segmented to delineate 105 anatomical structures. These structures were then registered (non-linearly to correct for spatial distortions) into a 3D coordinate space. Of the 105 delineated structures, approximately half correspond to thalamic nuclei, parcellated according to the nomenclature of Hirai and Jones (1989) and cross-referenced to the Schaltenbrand and Wahren (1977) labeling, spanning the anterior, mediodorsal, ventral, and pulvinar nuclear groups. This atlas remains one of the most commonly used reference systems for MRI-based *in vivo* neuroimaging studies.

Krauth et al. (2010) created a high-resolution 3D atlas of the human thalamus and its subdivisions by integrating digitized histological delineations from the Morel (2007) atlas. This approach relied on Morel’s multi-architectonic delineations. With the effort to overcome the anisotropic and topologically ambiguous nature of single histological stacks, Krauth et al. (2010) iteratively registered and averaged multiple stereotactically oriented histological maps to generate an unbiased probabilistic 3D representation of thalamic nuclei and their interindividual variability. Inter-individual variability has been represented in the form of statistical shape models. By rendering histological detail in a reproducible 3D reference space (Iglesias et al., 2018), the atlas has been applied in functional imaging studies (Hwang et al., 2017; Wang et al., 2025) and neurosurgical planning (Accolla et al., 2016; Horn et al., 2019).

The BigBrain offers a high-resolution 3D reconstruction of the human brain. It is based on 7404 microscopic histological data from one paraffin embedded brain, stained for cell bodies using the Modified Merker method (Amunts et al., 2013). This ultrahigh-resolution dataset, with 20 μm isotropic voxel spacing, allows visualization of fine structures, such as cortical layers and subcortical nuclei. While the BigBrain histological template itself represents the thalamus as a unified structure, it has been crucial in integrating 2D histological atlases into a 3D framework with higher spatial resolution compared to previous atlases (Pascal et al., 2018). The multimodal, probabilistic Julich-Brain Atlas integrates high-resolution cytoarchitectonic maps with microstructural and connectivity data as well as neurotransmitter receptor expression profiles and functional data (Amunts et al., 2020). By incorporating information from multiple brains probabilistically, it captures inter-individual variability in the size, shape, and location of anatomical structures. This provides a framework for integrating multi-level data, including histology, receptor architecture, connectivity, and function, and is used in studies of complex regions such as the thalamus and basal ganglia, enabling precise mapping of structure–function relationships, interindividual variability, and applications in neuroimaging, computational modeling, and neurosurgical planning. Combining this probabilistic, multi-brain framework with BigBrain’s ultrahigh-resolution single-subject template has enabled detailed parcellation of metathalamic nuclei beyond what either resource offers alone; for example, subdivisions of the medial geniculate body (dorsal, medial, ventral) and the six-layered lateral geniculate body, mapped bilaterally at microscopic resolution, offering novel insight into thalamic organization (Kiwitz et al., 2022).

The human motor thalamus atlas (Ilinsky et al., 2018) was developed to provide a stereotactic atlas of the human motor thalamus using serial sagittal Nissl-stained section from a single post-mortem human brain. To complement the cytoarchitectonic delineation, the authors included immunohistochemistry for glutamic acid decarboxylase isoform 65 (GAD65), a histochemical marker that labels afferent terminals from the basal ganglia and local circuit interneurons (Kultas-Ilinsky et al., 2011). In pallidal and nigral territories, GAD65 directly marks inhibitory afferent terminals, as these projections are themselves GABAergic; in the cerebellar territory, where afferents are excitatory, GAD65 instead reveals the local interneuron network. Together, these patterns enabled identification of motor thalamic territories. Individual nuclei were segmented throughout the histological slices and reconstructed into a 3D stereotactic atlas. This was initially referenced to the Talairach stereotactic coordinate system relative to the IC line and then co-registered to MNI-152 space, making the atlas integrated in standard neuroimaging workflows. Unlike atlases based primarily on cytoarchitectonic criteria, this connectionally informed parcellation provides an anatomically meaningful framework to identify functionally relevant motor thalamic subregions, making it valuable for DBS targeting and other stereotactic neurosurgical applications.

Most recently, Alkemade et al. (2022) developed an open source, 3D whole brain atlas combining multiple microscopy stains and a 7T quantitative MRI. The atlas provides whole-brain data and reconstructions of sections labeled using five different immunohistochemical procedures, including Nissl and silver staining, parvalbumin, calretinin and calbindin staining. All histological data were reconstructed in a common block-face coordinate space and co-registered with 7T quantitative MRI at 200 μm resolution, providing a multimodal, 3D representation of both microstructural and macroscopic anatomy. The thalamus has already been parcellated using various *in vivo* MRI contrasts, but discrepancies exist between different approaches. This atlas provides immunohistochemical reference boundaries that can be compared with MRI contrast variation. Mapping microscopy-derived labels onto MRI enables assessment of nucleus-specific MRI differences and supports the development and validation of automated thalamic segmentation methods.

## 3 MRI based thalamus atlases

Atlases derived from specialized MRI sequences are increasingly important for neuroanatomical studies, as they provide non-invasive visualization of subcortical structures and enable optimal contrast for delineating thalamic substructures. Supplementary Table 1 summarizes the key MRI atlases, including the MRI acquisition field strength, sequences, and the specific nuclei delineated. In contrast to the previous chapters, we focus on those atlases that primarily used MRI data for structural delineations. Many studies rely on optimized MRI sequences to enhance thalamic contrast, therefore, many of the visualized nuclei listed in Supplementary Table 1 are largely based on qualitative assessment rather than ground truth or histology.

As one of the first openly available atlas, the thalamic connectivity atlas developed by Behrens et al. (2003) represents one of the first attempts to map human thalamocortical organization using diffusion-weighted (DW) imaging. Their approach involved acquiring DWI data from 8 healthy volunteers, after which the cerebral cortex was parcellated into large, anatomically defined regions (prefrontal, motor, somatosensory, parietal, temporal, and occipital cortices). Using a probabilistic tractography algorithm, they estimated the probability of connection between individual thalamic voxels and these cortical regions, which was the basis of the parcellation of the thalamus into subregions defined by their dominant cortical connections. These subdivisions have functional relevance, that well aligns with known thalamic nuclear divisions. As their method uses “structural connectivity” instead of cyto- or myeloarchitecture, it reveals connectional patterns of thalamic organization, which is complementary to the “classical,” histology-based atlases.

Jakab et al. (2012) introduced a novel hybrid workflow for constructing a probabilistic thalamic atlas by combining statistical shape models (SSMs) with DTI connectivity information. DTI from 40 subjects were mapped to standard space and combined with a statistical shape model trained on 3D reconstructions of 7 histological thalami based on the Krauth/Morel delineations and their digitized versions (Morel, 2007; Krauth et al., 2010). The method therefore integrated outline-based anatomical priors with DTI-derived internal connectivity landmarks to predict thalamic nuclei, providing a spatial reference, and a way to “individualize” thalamus maps, if DTI data is available. Using SSM-based alignment, Jakab et al. (2024) transformed the Morel atlas into MNI152 space. The resulting anatomical label maps are available in NIfTI (.nii.gz) format, co-registered to the MNI152 T1 template at 1 mm and 0.5 mm resolution.

Najdenovska et al. (2018) introduced an *in vivo* probabilistic thalamic atlas derived from diffusion weighted MRI. Their approach builds on diffusion based segmentation methods, such as the one described in Battistella et al. (2017), which use microstructural features to define thalamic subdivisions. Using publicly available Human Connectome Project data, the authors developed a clustering based parcellation of the thalamus in a common space. Their atlas represents diffusion informed subdivision and can be used as a reference when subject specific diffusion MRI is not available, although it does not replace individualized diffusion-based segmentation.

The probabilistic atlas of Iglesias et al. (2018) integrates *ex vivo* MRI and histology. Six postmortem brains were first scanned on a 3T MRI, followed by sectioning of the thalamic tissue blocks at 50 μm intervals. One in every ten sections was processed using Nissl staining. Their atlas includes detailed cytoarchitectonic analysis and the manual delineation of 26 individual thalamic nuclei across all specimens. Histological segmentations were combined with manual delineations from *in vivo* T1-weighted MRI (39 subjects), with 3D reconstruction achieved by aligning serial histological sections to *ex vivo* MRI using blockface images to correct distortions. Applied to *in vivo* data with Bayesian segmentation, the atlas shows high test–retest reliability, robustness across MRI contrasts, and sensitivity to thalamic changes in Alzheimer’s disease. Most recently, Liu et al. (2022) developed and validated a reproducible diffusion MRI tractography protocol, reconstructing white matter tracts at the level of individual thalamic nuclei and their cortical targets, demonstrating high computational and test-retest reproducibility (Mengxing et al., 2023). Applying this pipeline with thalamic segmentation derived from the Iglesias et al. (2018) probabilistic atlas, they reconstructed 39 distinct tract pairs connecting the mediodorsal nucleus with specific prefrontal cortical areas, showing the increasing anatomical granularity achievable with modern DWI-based approaches.

Poor tissue contrast on conventional T1-weighted MRI has limited reliable *in vivo* delineation of thalamic substuctures. Su et al. (2019) introduced THOMAS (Thalamus Optimized Multi-Atlas Segmentation), a fully automated multi-atlas segmentation framework to delineate 12 thalamic nuclei from white matter-nulled MP-RAGE images. The method demonstrated high agreement with expert manual segmentations while substantially reducing segmentation time. The authors validated THOMAS on datasets from healthy volunteers and patients with multiple sclerosis, using volumetric analysis to identify nucleus-specific atrophy. The authos also demonstrated the clinical utility of THOMAS for surgical planning by evaluating its ability to localize the ventralis intermedius nucleus (Vim), a key deep brain stimulation target for essential tremor. Their results showed good correspondence between THOMAS derived Vim boundaries and the actual location of the tissue and DBS contracts in the treated patients.

Saranathan et al. (2021) developed an *in vivo* structural MRI atlas of the human thalamic nuclei, building on the improved contrast of white matter–nulled MP-RAGE sequence. They used 9 scans acquired at a spatial resolution of 0.7 × 0.7 × 0.5 mm^3^, with thalamic nuclei manually segmented using the Morel atlas as a histological reference for visual identification and nomenclature of thalamic nuclei, and as a spatial benchmark for validating MRI-derived nucleus localization in a common coordinate space (Tourdias et al., 2014). A group template was generated through iterative averaging and nonlinear warping of the individual datasets, which was subsequently registered to MNI space. The resulting atlas provides both spatial probability maps and maximum probability maps of the thalamic nuclei in template space at high resolution. The authors validated the atlas on white matter-nulled data, as well as conventional MP-RAGE and 7T acquisitions using independent datasets from healthy participants and from patients with multiple sclerosis and essential tremor. This demonstrated that their atlas retains utility beyond the specialized sequenced used in its original construction, and thus broadening its applicability to more widely available clinical and research imaging protocols.

### 3.1 MRI sequences and segmentation algorithms for thalamic nuclei visualization

T1 and T2 weighted MRI sequences used in standard clinical imaging provide insufficient tissue contrast to delineate individual thalamic nuclei (Saranathan et al., 2021). Advances in MRI acquisition have focused on improving intra-thalamic contrast for direct visualization. At ultrahigh-field MRI (7T and 9.4T), and with optimized protocols at clinical field strengths (1.5T and 3T), higher-resolution imaging of thalamic architecture has become feasible. White-matter nulled (WMn) MPRAGE sequences improve the visualization of thalamic nuclei boundaries at both 3T and 7T (Tourdias et al., 2014; Su et al., 2019; Liu et al., 2020; Saranathan et al., 2021; Pfefferbaum et al., 2023). These sequences use the differences in myelin content across thalamic nuclei and laminar boundaries, which results in a better intra-thalamic contrast.

Quantitative Susceptibility Mapping (QSM) relies on variations in magnetic susceptibility, enabling visualization of nuclei with distinct iron and myelin content. QSM visualizes iron-rich thalamic nuclei like the anterior nucleus, mediodorsal nucleus, and pulvinar as high-contrast regions at 3T (Harada et al., 2022). At ultrahigh field (9.4T), QSM has shown nucleus-specific patterns of paramagnetic and diamagnetic sources in the thalamus and basal ganglia (Kumar et al., 2021).

High-resolution proton-density weighted (PDw) MR imaging sequences has also been shown to directly selected thalamic nuclei, especially the centromedian (CM) complex and other densely packed nuclei (e.g., LGN or thalamic reticular nucleus) is surrounded by axons and dendrites (Kanowski et al., 2010). Later work has replicated PDw utility for selected intrathalamic targets (Viviano and Schneider, 2015).

Diffusion MRI based methods are emerging for thalamus visualization, for example, by inferring thalamo-cortical connectivity patterns as the source of contrast (Sedrak et al., 2011; Kumar et al., 2015; Battistella et al., 2017). Using probabilistic tractography, Behrens et al. (2003) demonstrated that the thalamus could be segmented based on their predominant cortical connections. Battistella et al. (2017) described a novel approach by using information from the orientation distribution functions (ODFs) reconstructed in a spherical harmonics basis from diffusion MRI at 3T as features for thalamus subdivision. Opposed to the long-range connectivity method, this uses only localized information, and it was found to be sufficiently robust and major subdivisions of matching histological atlases.

When direct visualization remains limited, particularly at standard clinical field strengths, segmentation approaches can support thalamic parcellation. Deoni et al. (2007) introduced a segmentation method relying on the differences in quantitative T1 and T2 relaxation times to distinguish major thalamic nuclei. This method was later refined by Traynor et al. (2011), optimizing segmentation parameters, revealing that the best results balanced T1 and T2 dependence with spatial priors. However, these approaches still do not provide enough tissue contrast for subdividing larger nuclei into sub-nuclei described in histological atlases.

### 3.2 Free and open-source software for visualizing and navigating the thalamus

FSL (FMRIB Software Library, RRID:SCR_002823) (Smith et al., 2004; Woolrich et al., 2009; Jenkinson et al., 2012) provides tools for the analysis of structural and diffusion MRI data, including a connectivity-based thalamus atlas derived from DTI. Thalamic segmentation is implemented in the FSL-FIRST module, which enables extraction of thalamic volumes and assessment of shape and connectivity.

Freesurfer (RRID:SCR_001847) (Fischl, 2012) includes thalamic nuclei segmentation through the ThalamicNuclei based on the Iglesias et al. (2018) atlas. This approach requires a T1 scan processed through recon-all, or an additional scan of a different modality, showing better contrast between the nuclei. A diffusion-informed variant, ThalamicNucleiDTI incorporates information from diffusion imaging to refine parcellation (Tregidgo H. et al., 2023; Tregidgo H. F. J. et al., 2023).

Lead-DBS (RRID:SCR_002915) (Horn and Kühn, 2015, p. 201; Horn et al., 2019; Neudorfer et al., 2023) provides a framework provides a framework for image registration, electrode localization, and atlas-based visualization in the context of deep brain stimulation. Lead-DBS has pre-installed set of atlases, including subcortical atlases such as the Allen human reference atlas (Ding et al., 2016), Human Motor Thalamus atlas (Ilinsky et al., 2018), THOMAS Atlas (Su et al., 2019), and the digitalized version of the Morel thalamus atlas (Morel, 2007; Krauth et al., 2010; Jakab et al., 2012).

VolBrain (RRID:SCR_021020) (Manjón and Coupé, 2016) performs automated brain volumetry, including thalamic segmentation via the DeepThalamus module. This method relies on the Morel stereotactic atlas as a reference (Ruiz-Perez et al., 2025) and uses a multimodal deep neural network to achieve fine-grained parcellation.

## 4 Conclusion

The evolution of brain atlases has progresses from early hand-drawn maps to high-resolution 3D reconstructions and multimodal digital resources. In the thalamus, this development has enabled detailed characterization of its nuclear organization. Histology-based atlases have been the reference standard for nuclei delineation, despite limitations related to tissue distorition and interindividual variability. MRI-based atlases, on the other hand, provide reliable spatial reference for navigation *in vivo*. Hybrid atlases that combine histology with *ex vivo* or *in vivo* MRI have the potential to bridge the gap between cellular-level information and clinical utility.

Beyond structural delineation, recent methods have used quantitative MRI contrasts, diffusion tractography, and functional MRI to reveal nuclei-specific differences in myelination, iron content, and connectivity. Such approaches complement histology and allow for subject-specific thalamic parcellation *in vivo*. These methods may facilitate more precise targeting in applications such as deep brain stimulation targeting, mapping disease-related atrophy, and detecting subtle network alterations in neurodegeneration. Open-source pipelines and probabilistic frameworks have increased accessibility to thalamic segmentation tools across both research and clinical domains.

Thalamic segmentation is further complicated by inconsistencies in nomenclature and parcellation schemes across atlases. Mai and Majtanik (2019) compared nine widely used thalamic atlases and highlighted discrepancies in naming conventions and in the delineation of specific subregions. These differences likely reflect underlying neuroanatomical traditions and methodological choices and contribute to variability in cross-study comparisons. Harmonization of nomenclature has therefore been proposed to improve comparability across datasets.

This need for harmonization has also been emphasized by Segobin et al. (2024), on behalf of the Thalamus Nuclei Neuroimaging Group (TANGO) consortium. They propose a six-step framework: establishing thalamic anatomical “ground truths” through expert consensus; harmonizing nomenclature; defining best-practice MRI acquisition protocols; developing standardized segmentation algorithms and toolboxes; building large-scale open-source datasets; and publishing formal guidelines via an international Delphi consensus process. They argue that changes in the thalamic nomenclature across histological atlases since the 1970s have made it difficult to accurately co-register histology-based atlases and modern MRI data. Notably, the first step of the roadmap of establishing thalamic “ground truths” regarding nuclear number, location, and nomenclature depends on resolving the historical and conceptual differences between existing atlas frameworks. This represents a key point of convergence with the present review, which describes how methodological advances, from classical cytoarchitecture to stereotactic and multimodal imaging approaches, have shaped current thalamic atlases. Segobin et al. (2024) provide an application-oriented roadmap focused on improving the standardization and implementation of MRI-based thalamic segmentation, and this review provides the historical and methodological context required to understand why such standardization remains challenging. This highlights the need to integrate anatomical knowledge from established histological atlases with modern neuroimaging frameworks to achieve reliable and comparable thalamic parcellation.

Despite the large number of existing brain atlases, their diversity reflects differences in methodology rather than redundancy. Developments in atlas construction have improved the structural localization and cytoarchitectonic detail by increasing spatial resolution, refining boundaries, and capturing interindividual variability. For example, the 3D mean atlas of the human thalamus by Krauth et al. (2010) incorporated multiple histological stacks to generate a refined average model of thalamic subdivisions. More recently, a probabilistic atlas combining *ex-vivo* MRI and histology delineated 26 thalamic nuclei showed improved precision and reliability (Iglesias et al., 2018). Classical staining techniques such as Nissl and myelin staining remain widely used due to their reproducibility and comparability across studies, although their continued dominance may limit integration with newer imaging modalities. Current approaches increasingly combine histological methods with ultra-high field MRI, transcriptomics, and connectomics to provide more comprehensive characterizations of brain architecture.

Large-scale initiatives such as the BRAIN Initiative aim to develop cellular-resolution atlases through the integration of molecular, anatomical, and physiological annotations of cell types (Ecker et al., 2017). These datasets provide increasingly detailed descriptions of how neuronal populations are distributed and interconnected, extending beyond macroscopic brain atlases. In addition to their relevance for basic neuroscience, they support the development of more biologically constrained *in silico* models.

Neuronal morphology data, including dendritic arborization, axonal projections, and synaptic distributions, can improve the anatomical realism of computational models and enable the simulation of emergent network properties such as oscillations, synchronization, and state-dependent dynamics. As demonstrated by Iavarone et al. (2023) in the reconstruction of morpho-electrical models of the thalamoreticular circuit in various brain states by utilizing 3D neuron morphology data from the MouseLight Project (Winnubst et al., 2019). It should be noted that both of these studies are based on rodent circuits. Their relevance to the present discussion of human thalamic atlases lies not in direct species-level applicability, but in the methodological principle they illustrate, namely that anatomically detailed atlases provide the structural substrate required to build biologically constrained *in silico* models. A further example of this principle is Sheiban et al. (2026) who developed a data-driven 3D anatomical scaffold model of mouse motor thalamic nuclei to simulate spindle oscillations, illustrating how atlas-derived spatial constraints can ground circuit-level simulation regardless of species.

Looking ahead, the integration of multi-scale data, ranging from cytoarchitectonics to quantitative MRI and computational models may help align differences in parcellation schemes and improve consistency across thalamic atlases. The thalamus, as a central hub of cortico-subcortical communication, stands to benefit from these developments. By linking structure to function across scales, next-generation atlases may refine our understanding of thalamic organization and support clinical interpretation, including applications in diagnosis, treatment planning, and monitoring of neurological and psychiatric disease.
