# Supplementary material for: Mapping the human thalamus: a historical review of thalamic atlases from histology to modern neuroanatomy
Source: Front Neuroanat. 2026 Aug 10;20:1927141. doi: 10.3389/fnana.2026.1927141 (PMC13530975; doi:10.3389/fnana.2026.1927141)
Supplement: Supplementary file 1 [file Data_Sheet_1.docx]

Supplementary Material

# 1 Supplementary Tables

**Table S1.** MRI studies enabling intra-thalamic nuclei delineation: acquisition sequences, field strengths, and reference atlases.

| **Author** | **Field Strength (T)** | **Sequence** | **Reference atlas** | **Visualised nuclei** |
| --- | --- | --- | --- | --- |
| **Deoni et al., 2007** | **1.5** | **DESPOT1/DESPOT2** | **Talairach** | **MD, CM, CL, LD, LP, VA, VLa, VLPd, VLPv, VM, VPM, VPL, *Pul*, LGN, MGN** |
| **Yovel & Assaf, 2007** | **3** | **Multiple** | **Talairach** | **Pul, MD, LP, LD, VPL VPM, VL, VA, A** |
| **Gringel et al., 2009** | **3** | **multiecho FLASH** | **Hirai + Jones** | **LVtr, MdTh, LaTh, PyTr** |
| **Sudhyadhom et al., 2009** | **3** | **FGATIR** | **S+B** | **Voa, Vop, Vim, Vc** |
| **Kanowski et al., 2010** | **3** | **MPRAGE, PDw turbo-spin-eho** | **Hassler** | **CM** |
| **Bender et al., 2011, p. 20** | **3** | **MPRAGE** | **S+W** | **AV, VA, Vla, VLp, VPL, CeM, MD, CL, LP, PuM** |
| **Traynor et al., 2011** | **3** | **Quantitative T1 and T2 maps** | **Morel** | **PuM, VLPv, MD, LP, VLPd, PuL, AV, VA, VPL, Pf, Vla, CM, VM, VPM** |
| **Vassal et al., 2012** | **1.5** | **WAIR** | **Morel, S+W** | **Vim, M, DM, DL, I, Vo, pfF, VCM, VCL, CM, Pf, Pul, AL, Am** |
| **Deistung et al., 2013** | **7** | **Quantitative SWI** | **Morel, S+W** | **A.pr, Ce, D.c, D.im, D.o, Fa , F.M, La.m.ip, La.m.o, Li, Lpo, M Pf, Pu, Pu.l, Pu.m, Pu.o, Pu.o.v, Pu.s.f, S.pv, St.t, T.mth, V.c, V.im, V.o.a, V.o.i, V.o.m., V.o.p, CL, LMM** |
| **Tourdias et al., 2014** | **7** | **White-matter nulled MPRAGE** | **Morel** | **LP, MD, CM, PuA, VPL, RN, LGN, VA, VLA, VLP, MTT, CeM, PuM, PuL, AV** |
| **Zerroug et al., 2016** | **1.5** | **WAIR** | **-** | **Med, IL, DL, Cm, Vcm, Pf, PLR, AL, Vo, Am, Vim, Mtb** |
| **Battistella et al., 2017** | **3** | **T1-weighted MPRAGE** | **Morel** | **A, VA, MD, VLV, VLD, Pul, CL-LP-PuM** |
| **Iglesias et al., 2018** | **3** | **3D multi-slab balanced stead-state free precession** | **-** | **AV, LD, LP, VA, VAmc, VLa, VLp, VPL, VM, CeM, CL, Pc, CM, Pf, Pt, MV-re, MDm, MDl, LGN, L-SG, PuA, PuM, PuL, PuI, R** |
| **Su et al., 2019** | **7/3/1.5** | **WMn MPRAGE** | **Morel** | **AV, CM, MD-Pf, MGN, MTT, Pul, VA, VLP, Vla, VPL** |
| **Corona et al., 2020** | **3** | **MPRAGE** | **Morel** | **L, M, P** |
| **Liu et al., 2020** | **7** | **T1-weighted MPRAGE / SWI** | **Morel** | **AVD, AM, LD, MD, CeM, Pv, CM, Pf, CL, Hb, VA, Vla, VLp, VPL, VPM, VPI, VM, LP, PuMI, PuA, PuL, Li, MTT** |
| **Basile et al., 2021** | **3** | **MPRAGE** | **-** | **A, CM, Hb, LD, LG, MD/CL, MG, Ml, Pul, VA, VL, VP** |
| **Saranathan et al., 2021** | **7** | **WMn MPRAGE** | **Morel** | **AV, VA, VLa, VLp, VPl, Pul, LGN, MGN, CM, MD-Pf, Hb, MTT** |
| **Kumar et al., 2021** | **9.4** | **MP2RAGEE, GRE** | **Morel** | **MGN, VPI, VPL, VPM, VL, CeM, CL, CM, PuA, Pul, PuL, PuM, AD, AM, AV, MD** |
| **Pfefferbaum et al., 2023** | **3** | **WMn MPRAGE/T1-weighted SPGR** | **Morel** | **AV, VA, Vla, VLp, VPl, Pul, LGN, MGN, CM, MD, Hb** |
| **Charyasz et al., 2023** | **3/9.4** | **MPRAGE** | **Iglesias** | **AV, LP, VA, Vla, VLp, VPL, PuM, PuA, PuI, PuL, Pf, CM, CeM, MDm, MDl** |

**DESPOT1/DESPOT2: Driven Equilibrium Single Pulse Observation of T1 and T2, MPRAGE: Magnetization-prepared rapid gradient-echo, FGATIR: Fast Gray Matter Acquisition T1 Inversion Recovery, PDw: Proton Density weighted imaging, SWI: Susceptibility weighted imaging, WMn: White-matter nulled, WAIR: White matter attenuated inversion recovery, MP2RAGE: Magnetization Prepared 2 Rapid Acquisition Gradient Echoes, GRE: Gradient Echo, SPGR: Spoiled Gradient Recalled, S+W: Schaltenbrand & Wahren Atlas. S+B: Schaltenbrand & Bailey Atlas. A list of the abbreviations for the thalamic nuclei can be found in the supplementary table S1.**

**Table S2.** Thalamic nucleus abbreviations used across MRI studies: anatomical groupings, full names, and nomenclature system equivalences

| **Group** | **Abbreviation** | **Full Name** | **Nomenclature** | **Notes** |
| --- | --- | --- | --- | --- |
| **Anterior group** | AD | Anterodorsal | Jones/Morel |  |
| **Anterior group** | AM | Anteromedial | Jones/Morel |  |
| **Anterior group** | AV | Anteroventral | Jones/Morel |  |
| **Anterior group** | AVD | Anteroventral dorsal | Morel | Subdivision of AV |
| **Anterior group** | A | Anterior nucleus (undivided) | Generic | Coarse grouping |
| **Anterior group** | AL | Anterolateral | Hassler/S+W |  |
| **Mediodorsal group** | MD | Mediodorsal (undivided) | Jones/Morel |  |
| **Mediodorsal group** | MDm | Mediodorsal medial part | Iglesias |  |
| **Mediodorsal group** | MDl | Mediodorsal lateral part | Iglesias |  |
| **Mediodorsal group** | MD/CL | Mediodorsal / Central lateral (merged) | Basile | Reported as single cluster |
| **Mediodorsal group** | MD-Pf | Mediodorsal / Parafascicular (merged) | Su / Saranathan | Reported as single cluster |
| **Ventral anterior group** | VA | Ventral anterior | Jones/Morel |  |
| **Ventral anterior group** | VAmc | Ventral anterior magnocellular part | Iglesias |  |
| **Ventral anterior group** | Voa | Ventral oral anterior | Hassler/S+W | ≈ VA in Jones |
| **Ventral anterior group** | Vop | Ventral oral posterior | Hassler/S+W | ≈ VLa in Jones |
| **Ventral anterior group** | Vo | Ventral oral (undivided) | Hassler/S+W |  |
| **Ventral anterior group** | V.o.a | Ventral oral anterior | Hassler/S+W | Same as Voa |
| **Ventral anterior group** | V.o.p | Ventral oral posterior | Hassler/S+W | Same as Vop |
| **Ventral anterior group** | V.o.i | Ventral oral intermediate | Hassler/S+W |  |
| **Ventral anterior group** | V.o.m | Ventral oral medial | Hassler/S+W |  |
| **Ventral lateral group** | VL | Ventral lateral (undivided) | Jones |  |
| **Ventral lateral group** | VLa | Ventral lateral anterior | Jones/Morel |  |
| **Ventral lateral group** | VLp | Ventral lateral posterior | Jones/Morel |  |
| **Ventral lateral group** | VLPd | Ventral lateral posterior dorsal part | Morel |  |
| **Ventral lateral group** | VLPv | Ventral lateral posterior ventral part | Morel |  |
| **Ventral lateral group** | VLD | Ventral lateral dorsal | Battistella | ≈ VLPd |
| **Ventral lateral group** | VLV | Ventral lateral ventral | Battistella | ≈ VLPv |
| **Ventral lateral group** | Vim | Ventral intermediate | Hassler/S+W | ≈ VLPv / VLp in Jones |
| **Ventral lateral group** | V.im | Ventral intermediate | Hassler/S+W | Same as Vim |
| **Ventral posterior group** | VP | Ventral posterior (undivided) | Generic | Coarse grouping |
| **Ventral posterior group** | VPL | Ventral posterolateral | Jones/Morel |  |
| **Ventral posterior group** | VPM | Ventral posteromedial | Jones/Morel |  |
| **Ventral posterior group** | VPI | Ventral posterior inferior | Jones/Morel |  |
| **Ventral posterior group** | VM | Ventral medial | Jones/Morel |  |
| **Ventral posterior group** | Vc | Ventral caudal | Hassler/S+W | ≈ VPL/VPM in Jones |
| **Ventral posterior group** | V.c | Ventral caudal | Hassler/S+W | Same as Vc |
| **Ventral posterior group** | Vcm | Ventral centromedian | Hassler/S+W |  |
| **Ventral posterior group** | VCM | Ventral centromedian | Hassler/S+W | Same as Vcm |
| **Ventral posterior group** | VCL | Ventral central lateral | Hassler/S+W |  |
| **Intralaminar group** | CM | Centre médian | All systems | Consistent across nomenclatures |
| **Intralaminar group** | CeM | Central medial | Jones/Morel | Distinct from CM |
| **Intralaminar group** | CL | Central lateral | Jones/Morel |  |
| **Intralaminar group** | CL-LP-PuM | Central lateral / Lateral posterior / Medial pulvinar (merged) | Battistella | Reported as single cluster |
| **Intralaminar group** | Pc | Paracentral | Jones/Iglesias |  |
| **Intralaminar group** | Pf | Parafascicular | Jones/Morel |  |
| **Intralaminar group** | IL | Intralaminar nuclei (undivided) | Generic | Coarse grouping |
| **Intralaminar group** | pfF | Parafascicular frontal part | Hassler |  |
| **Lateral group** | LD | Lateral dorsal | Jones/Morel |  |
| **Lateral group** | LP | Lateral posterior | Jones/Morel |  |
| **Lateral group** | L | Lateral group (undivided) | Generic | Coarse grouping |
| **Pulvinar group** | Pul | Pulvinar (undivided) | All systems |  |
| **Pulvinar group** | PuA | Anterior pulvinar | Morel/Iglesias |  |
| **Pulvinar group** | PuM | Medial pulvinar | Morel/Iglesias |  |
| **Pulvinar group** | PuL | Lateral pulvinar | Morel/Iglesias |  |
| **Pulvinar group** | PuI | Inferior pulvinar | Iglesias |  |
| **Pulvinar group** | PuMI | Pulvinar medioinferior | Liu et al. | Subdivision of medial pulvinar |
| **Pulvinar group** | Pu.l | Pulvinar lateralis | Hassler/S+W | ≈ PuL |
| **Pulvinar group** | Pu.m | Pulvinar medialis | Hassler/S+W | ≈ PuM |
| **Pulvinar group** | Pu.o | Pulvinar oralis | Hassler/S+W | ≈ PuA |
| **Pulvinar group** | Pu.o.v | Pulvinar oralis ventralis | Hassler/S+W | Subdivision of PuA |
| **Pulvinar group** | Pu.s.f | Pulvinar subfascicularis | Hassler/S+W |  |
| **Geniculate nuclei** | LGN | Lateral geniculate nucleus | All systems |  |
| **Geniculate nuclei** | LG | Lateral geniculate nucleus | All systems | Same as LGN |
| **Geniculate nuclei** | MGN | Medial geniculate nucleus | All systems |  |
| **Geniculate nuclei** | MG | Medial geniculate nucleus | All systems | Same as MGN |
| **Midline / paraventricular group** | MV-re | Reuniens (medioventral) | Iglesias | Midline nucleus |
| **Midline / paraventricular group** | Pv | Paraventricular | Morel |  |
| **Midline / paraventricular group** | Pt | Paratenial | Iglesias |  |
| **Midline / paraventricular group** | Ml | Midline nuclei (undivided) | Generic |  |
| **Midline / paraventricular group** | Med | Medial group (undivided) | Generic |  |
| **Midline / paraventricular group** | M | Medial (undivided) | Hassler/generic |  |
| **Reticular nucleus** | R | Reticular nucleus | Iglesias |  |
| **Mammillary-related landmarks** | MTT | Mammillothalamic tract | All systems | White matter tract used as anatomical landmark |
| **Mammillary-related landmarks** | Mtb | Mammillothalamic body | Zerroug et al. | Non-standard; likely MTT region |
| **Epithalamic nuclei** | Hb | Habenula | All systems |  |
| **Hassler / S+W specific** | A.pr | Anterior principal | Hassler/S+W |  |
| **Hassler / S+W specific** | Ce | Central (intralaminar) | Hassler/S+W |  |
| **Hassler / S+W specific** | D.c | Dorsal compact | Hassler/S+W |  |
| **Hassler / S+W specific** | D.im | Dorsal intermediate | Hassler/S+W |  |
| **Hassler / S+W specific** | D.o | Dorsal oral | Hassler/S+W |  |
| **Hassler / S+W specific** | Fa | Fasciculosus | Hassler/S+W |  |
| **Hassler / S+W specific** | F.M | Fibrae mediales | Hassler/S+W | White matter fascicle |
| **Hassler / S+W specific** | La.m.ip | Lamella medialis interna profunda | Hassler/S+W | Internal medullary lamina deep part |
| **Hassler / S+W specific** | La.m.o | Lamella medialis externa | Hassler/S+W | External medullary lamina |
| **Hassler / S+W specific** | Li | Limitans | Hassler/S+W |  |
| **Hassler / S+W specific** | Lpo | Limitans posterior | Hassler/S+W |  |
| **Hassler / S+W specific** | L-SG | Limitans / suprageniculate | Iglesias |  |
| **Hassler / S+W specific** | LMM | Lamella medialis medialis | Hassler/S+W | Internal medullary lamina medial part |
| **Hassler / S+W specific** | S.pv | Subparaventricularis | Hassler/S+W |  |
| **Hassler / S+W specific** | St.t | Stria terminalis (tract) | Hassler/S+W | White matter tract |
| **Hassler / S+W specific** | T.mth | Tractus mammillothalamicus | Hassler/S+W | MTT in other systems |
| **Other / mixed** | DM | Dorsomedial | Hassler/generic | ≈ MD in Jones |
| **Other / mixed** | DL | Dorsolateral | Hassler/generic | ≈ LP/LD in Jones |
| **Other / mixed** | I | Intralaminar (undivided) | Hassler |  |
| **Other / mixed** | PLR | Posterior lateral region | Zerroug et al. | Non-standard |
| **Other / mixed** | RN | Red nucleus | — | Not a thalamic nucleus |
| **Other / mixed** | Am | Anteromedial | Hassler/Vassal |  |
| **Other / mixed** | P | Posterior group (undivided) | Generic |  |
